# An integrated omics analysis: impact of microgravity on host response to lipopolysaccharide *in vitro*

**DOI:** 10.1186/1471-2164-15-659

**Published:** 2014-08-07

**Authors:** Nabarun Chakraborty, Aarti Gautam, Seid Muhie, Stacy-Ann Miller, Marti Jett, Rasha Hammamieh

**Affiliations:** US Army Center for Environmental Health Research Fort Detrick, 568 Doughten Drive, Fort Detrick, MD 21702-5010 USA

## Abstract

**Background:**

Microgravity facilitates the opportunistic infections by augmenting the pathogenic virulence and suppressing the host resistance. Hence the extraterrestrial infections may activate potentially novel bionetworks different from the terrestrial equivalent, which could only be probed by investigating the host-pathogen relationship with a minimum of terrestrial bias.

**Results:**

We customized a cell culture module to expose human endothelial cells to lipopolysaccharide (LPS). The assay was carried out onboard the STS-135 spaceflight, and a concurrent ground study constituted the baseline. Transcriptomic investigation revealed a possible immune blunting in microgravity suppressing in particular *Lbp*, *MyD88* and *MD-2*, which encode proteins responsible for early LPS uptake. Certain cytokines, such as IL-6 and IL-8, surged in response to LPS insult in microgravity, as suggested by the proteomics study. Contrasting proteomic expressions of B2M, TIMP-1 and VEGRs suggested impaired pro-survival adaptation and healing mechanisms. Differential expression of miR-200a and miR-146b suggested the susceptibility of hosts in spaceflight to oxidative stress and further underscored the influence of microgravity on the immunity.

**Conclusions:**

A molecular interpretation explaining the etiology of the microgravitational impact on the host-pathogen relationship elucidated comprehensive immune blunting of the host cells responding to LPS challenges. Longer LPS exposure prompted a delayed host response, potentially ineffectual in preventing pathogens from opportunistic invasion. Significant consequences include the subsequent failure in recruiting the growth factors and a debilitated apoptosis. Follow up studies with larger sample size are warranted.

**Electronic supplementary material:**

The online version of this article (doi:10.1186/1471-2164-15-659) contains supplementary material, which is available to authorized users.

## Background

During spaceflight, astronauts experience a unique set of stressors comprised of microgravity (μG), suboptimal nutrition, social isolation, atypical work environment, solar radiation, and alteration of circadian rhythm. Detrimental consequences were observed in the astronauts’ immune [1–3] and musculoskeletal systems [4], and many of the astronauts’ physiological [5] and cognition phenotypes [6, 7] were persistently altered long after the missions’terminations. The immunological investigations of the astronauts recorded several dysregulations, such as the altered production of cytokines [2], enhanced sympathetic neuroimmune responses [8], compromised functions of monocytes [9, 10], suppressed cytotoxicity of T-cells and Natural Killer (NK) cells [11], and reduced phagocytic capabilities of neutrophils [12]. A few aspects of immunological disorders such as the alteration of glucocorticoid-mediated immune response [8] were observed only after the long-term deployment for the space missions, which could be attributed to the accumulated effect of μG.

It has long been known that microorganisms such as *Escherichia coli* proliferate more rapidly in reduced gravity [13], thereby multiplying the risk of onboard cross-contamination, colonization, and infection. Worse, μG can potentially alter microbial physiology and augment pathogenesis as demonstrated by the studies using simulated μG [14, 15]. Together, host defenses under extraterrestrial stress could be highly susceptible to the opportunistic pathogens armed with their aggressive virulence and rapid proliferative aptitude [16].

A number of *in vitro*, *in vivo* and *ex vivo* studies probing space-flown biomaterials suggested a potential blunting of the immune response to pathogens and their various derivatives [16]. Of particular interest are the studies that investigated the impact of reduced gravity on the immunological responses to LPS shock. LPS, a common outer membrane component of typical gram-negative bacteria, can elicit strong immune responses in the host cells that may lead to sepsis [17, 18]. The serological responses of the astronauts were governed by the duration of the terrestrial LPS exposure to the whole blood samples *in vitro*. The shorter LPS exposure caused a prolonged elevation of interleukin-1ra (IL-1ra) accompanied by temporary elevations of IL-8 and LPS binding protein (LBP), and suppression of IL-6 and IL-1β [19]; while the longer LPS exposure suppressed the phagocytic activity and reduced the expressions of IL-6, IL-8, IL-1β and TNF-α that persisted for 7 d after the mission terminated [20]. The *in vitro* LPS exposure of spleen cells derived from space-flown C57BL/6j mice resulted in elevated *IL-6* and *IL-10*, but not *TNF-*α [21]. A rapid onset of LPS-induced apoptosis was observed in squids subjected to simulated μG [22].

To date, μG-induced immunological perturbations were investigated either by measuring the expressions of inflammatory markers in the space-flown biomaterials [3, 8, 9, 12, 1, 2, 10] or by presenting the space-flown host cells to the terrestrial endotoxic shock *in vitro*[19–21]. The expression analysis of astronauts’ serological markers [3, 8, 9, 12] was limited by the delay time between the mission termination and the assay initiation, which can potentially be an important time window for the stressed cells to get readjusted to the terrestrial environment. Likewise, inducing the pathogenic shock to the space-flown samples on ground [19–21] risked the host-pathogen relationship being critically influenced by the terrestrial bias. These limitations were systematically minimized as we attempted to probe the extraterrestrial impact on the host-pathogen relationships with minimum terrestrial bias.

The purpose of the present study was to understand the impact of μG on the *in vitro* host immune response to LPS assault. Towards this objective, the endothelial cells primed in the bioreactors were exposed to LPS for 4 h and 8 h at two gravitational limits. The project was integrated and flown under the direction of DoD's Space Test Program. Associated signatures at the genomic and proteomic levels were analyzed. To our knowledge, this is a novel molecular-level approach to assess the host cells infected in spaceflight; although previous studies probed the molecular makeup of pathogens inoculated in the spaceflight [23, 24]. Recent efforts used modeled μG to investigate the molecular makeups of the host [22]. The complexity of spaceflight could never be captured, however, by any of such simulated paradigms [25–27].

## Methods

### Reagents, cells, aseptic conditions and hardware

This *in vitro* study used commercially available cell lines, so ethics is not needed.

Human dermal microvascular endothelial cells (HMVEC-dBL; Lonza, Walkersville, MD) were maintained in EGM-2MV growth medium (Lonza, MD) containing growth factors, antimicrobials, cytokines and 5% FBS (all purchased from Lonza, MD) at 37°C in a humidified atmosphere containing 5% CO_2_. To avoid phenotypic drift associated with decreasing expression of surface receptor molecules, HMVEC-dBL was not used beyond passage 7. Fibronectin and LPS from *E. coli* 055: B5 were obtained from Sigma Chemical Co. (St. Louis, MO).

We selected a human micro-vascular endothelial cell line because of the manifold involvement of this cell type in the wound healing cascade. At the onset of wound repair, these endothelial cells typically coordinate the recruitment of cytokines and growth factors at the site of injury and subsequently initiate communication with the leukocytes and other tissues to trigger the healing cascade [28, 29].

**Figure 1:**
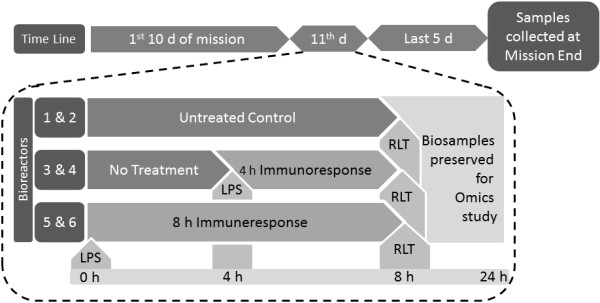
***Experiment scheme followed in the spaceflight and the concurrent ground study*** **.** Briefly, each of the two CCMs were loaded with six bioreactors. One CCM was operated in the spaceflight and the other one on ground, concurrently. Both CCMs followed same protocol depicted in the figure; where the top row shows the overall assay sequence segregated into three time zones: (i) the first 10 days, (ii) 11^th^ day and (iii) the last 5 days of the space mission. The protocol carried out on the 11^th^ day is elaborated inside the box bordered by the broken lines. The cells received typical nourishments during first 10 days of space mission. At the beginning of the 11^th^ day, 100 μg/ml LPS were injected to the flowpaths of bioreactors 5 and 6 in both CCMs (arrowhead with LPS 0 h). Four hours later, the same amount of LPS was injected into bioreactor 3 and 4 (arrowhead with LPS 4 h); and after the subsequent four hours, RLT solution was discharged, replacing the media in the flowpaths of all bioreactors [1–6] (arrowhead with RLT 8 h). The biosamples in the bioreactors [1–6] in both gravity limits were preserved in the RLT solution till the space shuttle landed 5 days after the RLT discharge. We collected the bioreactors 3 hours after the landing.

The Cell Culture Module (CCM) from Tissue Genesis, Inc., Honolulu, HI, is a state-of-art feedback controlled automated platform which was integrated and flown under the direction of DoD's Space Test Program. The CCM was employed with a few modifications to support the cell inoculations and their programmed treatment in spaceflight. This biocompatible module has been used in past space missions as a part of the Space Tissue Loss (STL) program [30]. In the present project, human endothelial cells were inoculated in Extra-Terrestrial Space (ECS), and solutions were injected through the Intra-Terrestrial Space (ICS) of the fibronectin-coated bioreactors following the customized protocol (Figure 1, Additional file 1: Figure S1).

### Protocol validations and final space mission

Cell Max (Spectrum Labs, Inving, TX), a semi-automated alternative of CCM was used to carry out the preliminary protocol optimization process (details in supplementary data) primarily to define the five following assay parameters. (i) One million cells was identified as the optimum cellular load per fibronectin-coated bioreactor. (ii) The treatment protocol outlined the exposures of 100 μg/ml LPS for 4 h and 8 h (Additional file 2: Figure S2). (iii) The RLT buffer (Qiagen, Germantown, MD) was selected as the suitable cell lysing agent and for subsequent preservation of the biomolecules (including RNA) for long periods of time at ambient temperature without causing any comprehensible degradation (Additional file 3: Table S1).

Figure 1 depicts the final experiment scheme, and the flowpaths of the CCM are shown in Additional file 1: Figure S1.

### Nucleic acid extraction from the bioreactors

The RLT solution was drawn from the the bioreactors using syringes, followed by washing with PBS. The bioreactors were inoculated with Trizol™ (Invitrogen, Inc., Grand Island, NY) to lyse any remaining non-denatured cells. We extracted mRNA and miRNA from the RLT solution using the AllPrep DNA/RNA extraction kit (Qiagen, MD) and from the Trizol™ portion following the manufacturer’s protocols. The nucleic acids were quantified and qualified by a NanoDrop ND-1000 spectrophotometer (NanoDrop Technologies, Wilmington, DE). The mRNA and miRNA (8.5 ng-12.5 ng) amplification was carried out using NuGEN Ovation Pico WTA system V2 kit (NuGEN Technologies, San Carlos, CA) and a Global miRNA amplification system (Systems BioSciences, Mountain View, CA) following the respective manufacturer’s protocol.

### mRNA whole genome oligonucleotide microarray

Dual dye microarray using the Whole Human Genome Microarray Kit (Agilent Technologies, Inc., Santa Clara, CA) was carried out following the vendor’s protocol. We labeled 12.5 ng amplified RNA with biotin and the same amount of reference RNA with fluorescein. Samples were hybridized to Agilent 4 × 44 k slides and incubated for 16 h at 55°C. A protocol with a series of washes was carried out for tagging Cy-3 and Cy-5 dyes to reference and sample RNA, respectively. The slides were scanned using Agilent Technologies Scanner G2505C US09493743 and feature extracted using the software v. 10.7 (Agilent, Inc., CA). The assay quality was verified by assessing no alteration of a set of housekeeping genes across the experimental parameters discussed in the supplementary section.

We have submitted the microarray data to the Gene Expression Omnibus (GEO) and this can be searched for using the GEO accession number: GSE54213.

### Statistical analysis and biological annotations

We used GeneSpring v.7.1 (Silicon Genetics, Redwood City, CA) to perform gene expression studies and clustering analysis. GraphPad Prism (GraphPad Software, Inc. La Jolla, CA) and R platform (http://www.r-project.org) were used for the statistical analysis. Unless otherwise mentioned, the analysis was performed using pair-wise moderate *t*-test with cut-off *p* < 0.05. It is a recommended routine for small sample populations to ensure maximum confidence scores [31].

Using the IPA platform, we mapped the regulatory networks significantly enriched with Genes of Interest (GoI). To construct the network module, IPA was used to mine the manually curated literature searches (Ingenuity® ExpertAssist Findings) and interaction data from third-party databases, such as IntAct, BIND. The multi-stage heuristic approach integrated the highly interconnected GoIs, and in the process, selected a more densely populated network over a sparsely populated alternative [32].

### Characterization of GoIs

The transcriptomic regulations responding to LPS treatments on the ground for 4 h and 8 h (defined as G-4 h and G-8 h, respectively) were normalized by the ground controls (G-C), potentially emphasizing *the host response exclusively induced by LPS*. Similarly, the transcriptomic regulations explaining the LPS-induced response mediated by μG for 4 h and 8 h LPS assault (defined as S-4 h and S-8 h, respectively) were normalized by their spaceflight baseline (S-C). Theoretically, the exclusive impacts of μG were suppressed only to highlight the gene’s interactive effects with the host responses to LPS (μG × LPS-induced host responses).

Nine pairs of biologically meaningful combinations were tested. The pairs, namely G-4 h vs. S-4 h and G-8 h and S-8 h were contrasted to understand the effect of μG on LPS assault. The pairs namely G-C vs. G-4 h, G-C vs. G-8 h, S-C vs. S-4 h and S-C vs. S-8 h were contrasted to understand the effect of LPS assault at two gravitation limits. The pairs namely G-4 h vs. G-8 h and S-4 h vs. S-8 h might illustrate the effect of durations at two gravitational limits. And, finally G-C vs. S-C might help in understanding the *exclusive* effect of μG.

The comparative analysis between the ground and space controls (G-C vs. S-C) revealed 2,517 transcripts (**Set A**; 6.3% of global gene set presented in the Agilent array). These genes are potentially the exclusive markers of μG.

A four hour LPS exposure carried out in two gravitational limits (G-4 h vs. S-4 h) altered 7,832 genes (**Set B**; 19.58% of global gene set presented in the Agilent array). This resulted in a potential set of markers explaining the extraterrestrial effects on the LPS-induced host response.

Among the other pairs of interest, 1,302 genes (**Set C**, 3.3% of global gene set presented in the Agilent array) emerged significantly different between 8 h LPS exposure in ground versus the controls inoculated in ground (G-8 h vs. G-C), a potential set of markers of the host response independent of the gravitational alteration.

All other pairs representing biologically meaningful combinations of interest including G-4 h vs. G-C, S-4 h vs. S-C, S-8 h vs. S-C, G-8 h vs. S-8 h, G-4 h vs. G-8 h and S-4 h vs. S-8 h failed to identify any genes significantly altered beyond the cut-off.

A Venn diagram (Additional file 4: Figure S3) illustrated the transcriptomic profile distributed among the three sets of genes defined hereby. **Set A** and **Set B** shared 1,534 genes likely to be manipulated by μG exclusively independent of any other factors including toxic shock. Similarly, there were 1,186 genes shared between **Set B** and **Set C** that could have shifted exclusively due to the host response independent of the gravitational shift. Screening off these potential false positive genes identified 5,379 genes (13.4% of global gene set presented in the Agilent array); defined as GoI-LPSμG, highlighting the *signatures of microgravitational impacts on LPS-induced host response* (μG × LPS induced host response).

Although the present study was primarily focused on understanding the impact of gravitational shifts on LPS-induced host responses, a parallel comparative investigation has been carried out to understand the *exclusive* impact of μG [1–3, 8–10, 12, 19–21]. Associated set molecular markers was constituted by 2,517 genes (Set A, defined as GoI-μG); 901 (36% of GoI-μG) and 1,616 (64% of GoI-μG) transcripts were up- and down-regulated in S-C in comparison to G-C.

### microRNA PCR assay

Selective real-time PCR assays were carried out using SABioscience kit (Qiagen, Inc.) using the amplified miRNA samples. As per the vendor’s protocol, we loaded 100 ng miRNA to 384-well plates containing anchored miRNA probes (50–75 bp; including miRNA sequence, tailing, and the universal primer) and the hybridization outcomes were quantified by the ABI HT 7900 real-time PCR system (Life technologies, Inc., Grand Island, NY). The vendor-recommended algorithm computed the relative miRNA expression level using the change of threshold cycle (Ct) *i.e.* 2 ^ (−Δ Ct), where Δ Ct = Ct (GoI) – avg. (Ct (HKG)). GoI represents the gene-of-interest, and HKG is the housekeeping gene. In order to eliminate the false positive candidates, the selected miRNA reads were screened by the following dual criteria applied together: (i) the control threshold cycle should be >30 and sample cycle <30 (or vice versa); and (ii) the *p*-value for the fold-change should be either unavailable or relatively high (p > 0.05) from the assay background.

From the pool of screened miRNA reads, we identified the probes that had different expressions (*p* < 0.05) between G-C vs. S-C. This cluster of miRNAs was altered *exclusively* by μG. Likewise, comparing G-4 h and S-4 h, the miRNA signatures of *LPS assault mediated by* μ*G* were identified.

### miRNA-mRNA target mining

We used the IPA platform (Ingenuity® Systems, http://www.ingenuity.com) to predict the mRNA targets of the selected miRNA modulators. The microRNA Target Filter predicted the mRNA targets by mining four databases, namely TargetScan, TarBase, miRecords, and the Ingenuity® Knowledge Base. The list was screened further to identify negatively correlated (r < −0.5) mRNA-miRNA pairs as described elsewhere [33].

### Immunoassay

The sump bags, dedicated to store the spent media at the end of mission, returned from space with 75 ml solution. Aliquots collected from the sump bags were centrifuged, and the cell-free supernatants were sent to Myriad RBM (Myriad RBM, Inc., Austin, TX) for Human Inflammation Multi-Analyte Profiling immunoassay. Assays were run on an automated Luminex MAP™ platform at the Myriad RBM CLIA certified lab and validated for the fundamental assay parameters of least detectable dose (LDD), lower limit of quantitation (LLOQ), spike recovery, linearity, precision and sample stability. Full assay validation documents are retrievable upon request from Myriad RBM (http://www.myriadrbm.com).

Complementary immunoassays were carried out in-house using either the high throughput multiplex the BioPlex immunoassay (BioRad, Hercules, CA) or using the 96-well format based sandwich ELISA. Both assay types were primarily carried out for validation purposes. For the BioPlex assay, all of the reagents were purchased from Millipore or Panomics (Affymetrix, Santa Clara, CA), and the results were analyzed using BioPlex manager software v4.1 (BioRad,. For the 96-well ELISA assay, we purchased the antibodies and other reagents from QIAGEN, Inc., R & D systems, and BD Biosciences. Each sample was assayed in triplicate, accompanied by appropriate quality controls.

## Results

### Identification of genes of interest (GoI) from the whole genome oligonucleotide assays

The host-pathogen relationship in μG could be (i) *exclusively mediated by LPS*, independent of any other foreign stimulators including the gravitational shift or (ii) *exclusively mediated by* μ*G*, independent of any other factors including the endotoxic shock or (iii) mediated by the *interplay of the two above mentioned factors* (μG × LPS-induced host responses). Present interpretation of the stressor landscape is technically a methodological decision made by the authors; thereby other interpretational possibilities cannot be ruled out. Furthermore, the role of fourth mediator explained by the factors beyond the control of experimental regulations could not be trivialized. We presume however that the carefully supervised experimental setup and the appropriately placed control studies potentially limited the roles of the uncontrolled influences.

The aim of the present study was to find the molecular signatures associated with the host immune response specific to LPS assault mediated by μG (μG × LPS-induced host responses). Meeting this objective, we processed the microarray mRNA data set by heuristic statistical manipulations accompanied by a systematic selection and rejection scheme described in supplementary data. The final outcome was 5,379 transcriptomic signatures associated with the host response to LPS assault mediated by μG (13.4% of global gene set presented in the Agilent array) defined as GoI-LPSμG (Additional file 3: Table S2).

A parallel comparative investigation identified 2,517 transcriptomic signatures of μG *exclusively* (6.3% of global gene set presented in the Agilent array), which we named as GoI-μG. This list is comprised of 901 (36%) and 1,616 (64%) transcripts over- and under-expressed in the spaceflight control (S-C), respectively, as compared to transcripts expressed in the ground control (G-C) (Additional file 3: Table S2). GoI-μG and GoI-LPSμG were designed as two orthogonal gene sets.

### Characteristics of GoI-LPSμG

The transcriptomic perturbations caused by 4 h and 8 h LPS exposures in ground control (defined by G-4 h and G-8 h, respectively) were nearly identical. Of the GoI-LPSμG, there were 50% and 44% transcripts of GoI-LPSμG elevated, 40% and 38% transcripts suppressed, and 8% and 17% transcripts unchanged in G-4 h and G-8 h, respectively. We considered the ground control (G-C) as the baseline for analyzing G-4 h and G-8 h.

Comparing G-4 h and G-8 h, there were more transcripts left unperturbed by the LPS exposures for 4 h (85% of GoI-LPSμG) and 8 h (61% of GoI-LPSμG) carried out in the spaceflight (defined by S-4 h and S-8 h, respectively). We considered the spaceflight control (S-C) as the baseline for analyzing S-4 h and S-8 h. Merely 15% of GoI-LPSμG were regulated in S-4 h; and among them 3% were up- and 12% down-regulated from S-C. In S-8 h, there were 21% up- and 18% down-regulated in comparison to S-C.

#### Principal component analysis (PCA)

**Figure 2:**
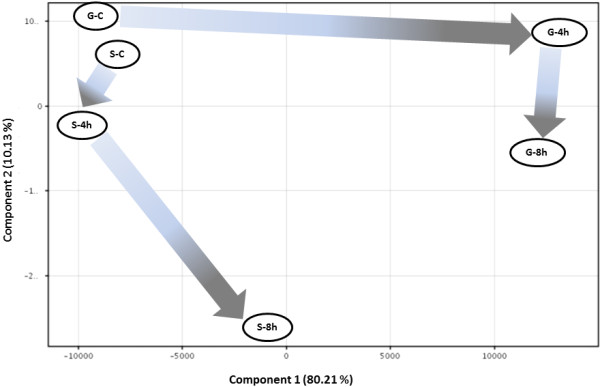
***Principal component analysis (PCA) of 5,379 transcriptomic signatures of host responses to LPS assault mediated by different gravitational forces.*** The arrows indicate the shifts of the transcriptomic expressions due to LPS insults at two gravitational limits. The genes were mined by pair-wise comparison between the LPS-treated samples normalized by the untreated samples inoculated on ground and in spaceflight respectively; for instance, G-4 h and G-8 h were normalized by G-C and S-4 h and S-8 h were normalized by S-C. The close Euclidian proximity of the self-normalized G-C and S-C implies that variance profile of this PCA does not represent the *exclusive* impact of microgravity. A distant clustering between the LPS assault carried out on ground (G-4 h and G-8 h) and that carried out in spaceflight, S-4 h in particular, is marked by PC 1 explaining 80% of total variance. Thereby the impact of microgravity on LPS assault emerges as the most significant factor in explaining the genomic perturbation among these 5,379 genes. Modest Euclidian proximity between S-4 h and S-C (r = 0.16) suggests an immune blunting in spaceflight. Clearly, the host in spaceflight failed to respond to LPS assault. The position of S-8 h equidistant from the two clusters formed by (a) S-C, G-C and S-4 h and to (b) G-4 h and G-8 h suggests a delayed host response in spaceflight.

The PCA (Figure 2) using GoI-LPSμG further explained the impact of μG on LPS-induced host responses. Principal components (PC) 1 and 2 explained 80.11% and 10.13% of the total variance, respectively, and together, they explained more than 90% of total variance.

### Significantly enriched immunologically relevant networks

Early signs of endothelial host response to LPS assault include activation of inflammatory signals, which results from the enrollment of growth factors and the modulation of apoptosis activities [34–36]. In this context, we trained the IPA platform to curate a set of pathways primarily focused on three families associated with (i) cytokines/chemokines, (ii) apoptosis, and (iii) growth factors (Additional file 3: Table S3). To note, several genes co-enriched more than one pathway. Likewise, several of the pathways were listed under more than one of the families; for instance, the GM-CSF pathway was listed under both cytokines/chemokines and growth factors, and here we documented it under the growth factors.

#### Cytokine signaling networks associated with GoI-LPSμG

**Figure 3:**
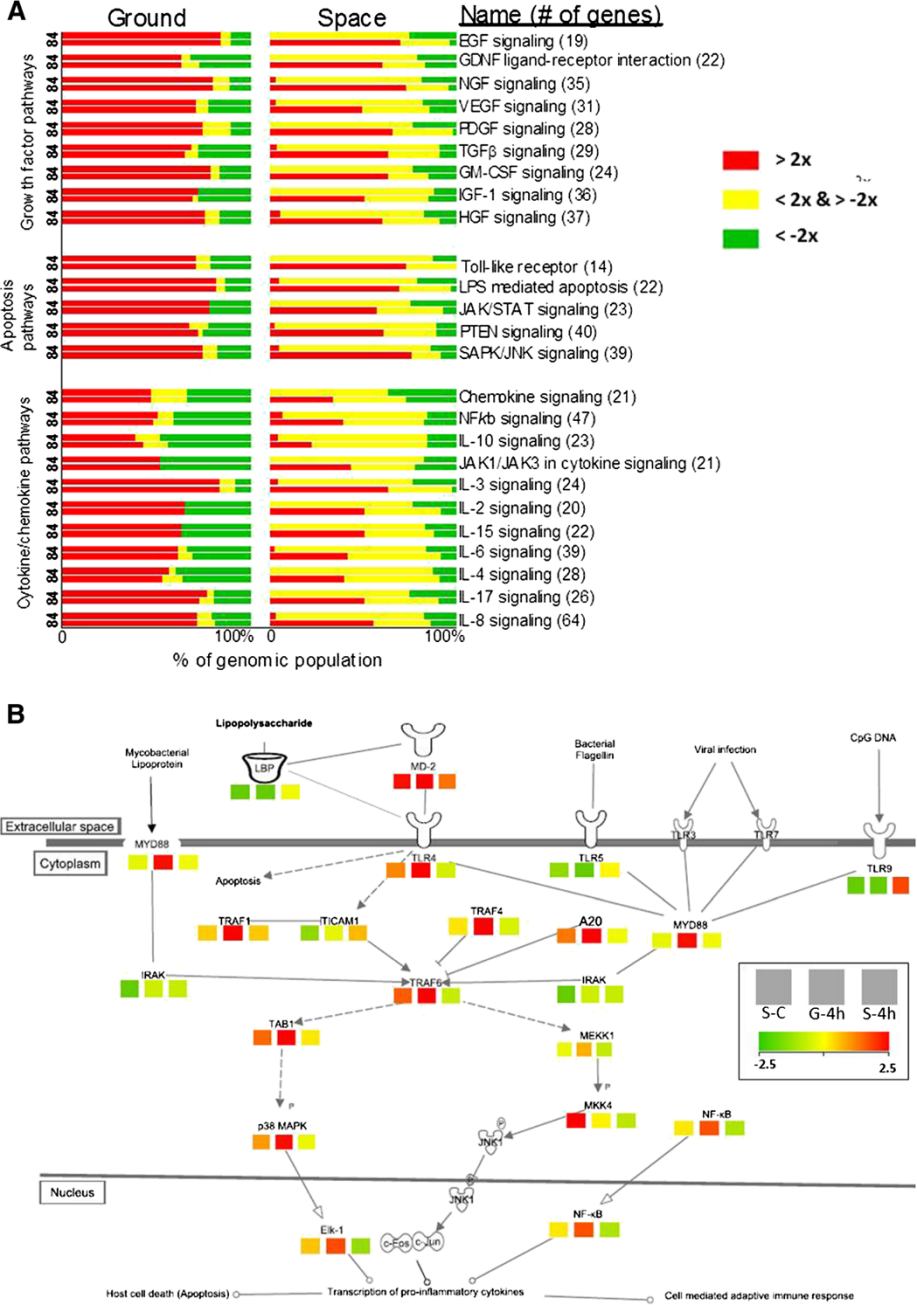
**Enrichment profile of the immunologically relevant pathways significantly altered by GoI-LPSμG with a focus on Toll-like receptor (TLR) pathway co-enriched by GoI-LPSμG and GoI-μG. A**. Enrichment profile of the immunologically relevant pathways significantly altered in responding to LPS assault mediated by different gravitational forces. Twenty four most significantly enriched pathways were identified focusing on three parent nodes, namely cytokine/chemokine pathways (11 networks) apoptosis pathways (5 networks) and growth factor pathways (8 networks). For each network, there are four bars segregated into two columns (Ground and Space) and two rows (4 h and 8 h, which are noted as 4 and 8, respectively). The distributions of genomic regulations associated with each pathway are hereby documented for G-4 h, G-8 h, S-4 h and S-8 h and the pathways are batched into three parent pathways: cytokine/chemokine pathways, apoptosis pathways and growth factor pathways. The fraction of the elevated (fold change > 2) and suppressed (fold change < −2) transcripts are colored by red and green, respectively. The unchanged fractions are yellow. The color scheme is at the right. **B**. *Toll-like receptors (TLR) pathway*. A schematic network of TLRs pathway is presented here. The TLRs pathway is significantly co-enriched by both gene sets linked to LPS insult mediated by microgravity (GoI-LPSμG) and exclusive effects of microgravity (GoI-μG), respectively. There are three blocks beneath each gene name representing the transcriptomic regulations of S-C (left most block), G-4 h (middle block) and S-4 h (right most block). G-8 h and S-8 h are omitted. The S-C regulations normalized by G-C (S-C/G-C) represent the exclusive impacts of microgravity. And, G-4 h and S-4 h normalized by respective controls (G-4 h/G-C and S-4 h/S-C) represent the influence of the gravitational shift on host response to LPS assault. The scale of the color scheme is included.

There were 160 genes identified from GoI-LPSμG significantly enriching 11 networks associated with the cytokine signaling (Figure 3A). These genes are documented in Additional file 3: Table S2 and their hierarchical clustering is shown in the Additional file 5: Figure S4B.

Interpreting the 160 genes, G-4 h and G-8 h shared high correlation (r = 0.96) enlisting 61% up- and 33% down-regulated transcripts. S-4 h with maximum number of transcripts (77% of 160 genes) unchanged from the S-C level was significantly different from rest of the three experimental conditions, namely S-8 h, G-4 h, and G-8 h (*p* < 0.0001, all three conditions). Demonstrating a modest correlation with G-4 h and G-8 h (r = 0.6, both conditions), S-8 h contained 41% of 160 transcripts showing S-C-like regulation.

#### Cytokine signaling networks associated with GoI-μG

There were 31 transcripts (34% up and 64% down-regulated) identified from GoI-μG significantly enriching 3 cytokine/chemokine signaling networks (Additional file 6: Figure S5). These genes are documented in Additional file 3: Table S2, and their hierarchical clustering is shown in the Additional file 5: Figure S4A. The IL-1 signaling network was significantly co-enriched by GoI-LPSμG and –μG (Additional file 7: Figure S6A).

#### Apoptosis signaling networks associated with GoI-LPSμG

**Figure 4:**
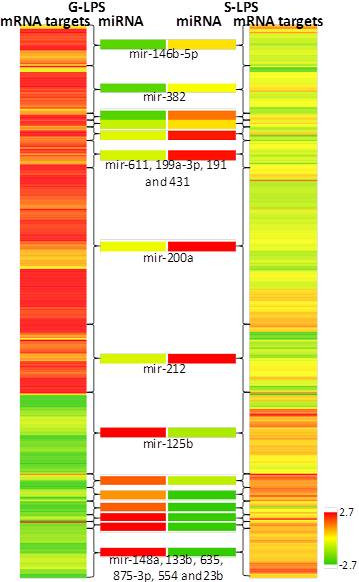
***miRNA signatures of host responses to LPS assault mediated by different gravitational forces and corresponding mRNA targets.*** Fifteen miRNAs significantly altered by 4 h LPS exposures carried out in two gravitational limits are clustered in two middle columns. There are 398 mRNAs collectively targeted by 15 miRNAs of interest. The four columns from left to right indicate the mRNA targets (1^st^ column) and corresponding miRNA probes (2^nd^ column), both regulated by the LPS assault in terrestrial gravity; miRNA probes (3^rd^ column) and corresponding mRNA targets (4^th^ column) both regulated by LPS assault in μG. The mRNAs are batched according to their miRNA modulators. The scale representing the color scheme is included.

There were 95 transcripts mined from GoI-LPSμG significantly enriching 5 apoptosis signaling networks (Figure 4). These genes are documented in Additional file 3: Table S2 and their hierarchical clustering is shown in the Additional file 5: Figure S4C.

Interpreting the 95 genes, G-4 h and G-8 h shared high correlation (r = 0.90), enlisting 75% up- and 20% down-regulated transcripts of the 95 genes. S-4 h had the maximum number of transcripts (79% of 95 genes) unchanged from the S-C level and was significantly different from rest of the three experimental conditions, namely S-8 h, G-4 h and G-8 h (*p* < 0.0001, all three conditions). Demonstrating significant difference from G-8 h (*p* < 0.05), S-8 h contained 26% transcripts of 95 genes showing S-C-like regulation.

#### Apoptosis signaling networks associated with GoI-μG

There were 28 transcripts (38% up- and 61% down-regulated) from GoI-μG significantly enriching 3 apoptosis signaling networks (Additional file 6: Figure S5). Identified genes are documented in Additional file 3: Table S2 and their hierarchical clustering is shown in Additional file 5: Figure S4A.

Of interest was the TLR signaling network, significantly co-enriched by GoI-LPSμG and –μG (Figure 4).

#### Growth factor signaling networks associated with GoI-LPSμG

There were 120 transcripts mined from GoI-LPSμG significantly enriching 8 growth factor signaling networks (Figure 4). These genes are documented in Additional file 3: Table S2, and their hierarchical clustering is shown in the Additional file 5: Figure S4D.

Interpreting the 120 genes, G-4 h and G-8 h shared high correlation (r = 0.95), enlisting 75% up- and 20% down-regulated transcripts of 120 genes. S-4 h had the maximum number of transcripts (79% of 120 genes) unchanged from the S-C level and was significantly different from rest of the three experimental conditions, namely S-8 h, G-4 h, and G-8 h (*p* < 0.0001, all three conditions). Demonstrating significant difference from G-8 h (*p* < 0.001), S-8 h contained 27% transcripts of 120 genes that showed S-C-like regulation.

#### Growth factor signaling networks associated with GoI-μG

There were 32 transcripts (44% up- and 56% down-regulated) mined from GoI-μG significantly enriching 4 growth factor signaling networks (Additional file 6: Figure S5). A list of these genes can be found in Additional file 3: Table S2 and the hierarchical clustering of the genes is shown in Additional file 5: Figure S4A.

Of interest was the GM-CSF signaling network (also featured under cytokine singling network), significantly co-enriched by GoI-LPSμG and GoI-μG (Additional file 7: Figure S6B).

### miRNA and corresponding mRNA targets

#### miRNAs associated with LPS treatment mediated by μG

**Figure 5:**
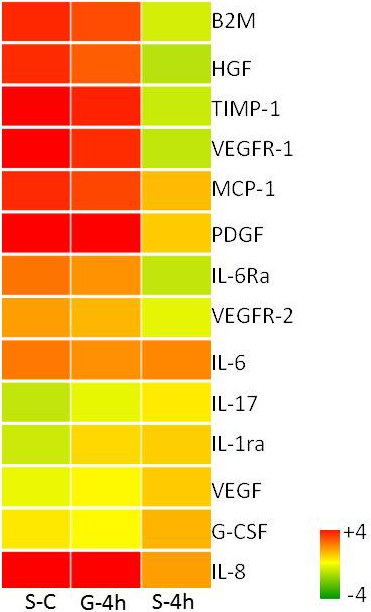
***Proteomics profile illustrating the impact of microgravity on the immune responses to LPS assault.*** The hierarchical clustering depicts the proteomic expression of the focused cytokine profile sampled from the 4 h LPS exposures carried out in two gravitational limits. The columns from left to right indicate the proteomic expression observed (i) in spaceflight compared to ground control (S-C/G-C)- marked as S-C, (ii) LPS exposure for 4 h compared to ground control (G-4 h/G-C)- marked as G-4 h and (iii) LPS exposure for 4 h compared to space control (S-4 h/S-C)- marked as S-4 h. The scale representing the color scheme is included.

From the pool of 110 miRNAs above the noise threshold, 15 miRNAs were significantly altered between S-4 h and G-4 h. Collectively, 15 miRNAs targeted 2,000 mRNAs from GoI-LPSμG. A subset of 398 mRNA targets were of particular interest, since their expression were negatively correlated (r < −0.5) with the expressions of their corresponding miRNA modulators [33]. A hierarchical clustering of the mRNA targets batched by corresponding miRNA regulators is shown in Figure 5.

A modest number of pathways associated with cytokine signaling, apoptosis, and growth factor were significantly enriched by 398 transcripts encoding the mRNA targets. More interestingly, the core of the mined networks still shared the same family trees enriched by the global gene set of GoI-LPSμG. The list included IL-15 signaling, HGF signaling, interferon signaling, IL-10 signaling, IL-6 signaling, TGF-beta signaling and TLR signaling.

miR-200a (fold change: G 4 h: −0.03; S-4 h: 1.13; r = − 0.67; *p* =0.09) regulated the highest number of transcripts (119), which was 30% of all the mRNAs collectively targeted by 15 miRNA.

#### miRNAs associated with μG

From the pool of 92 miRNA reads expressed above the noise threshold, nine were significantly different between the two gravitational limits. Collectively, these nine miRNAs targeted 600 mRNAs from GoI-μG. A subset of 169 mRNA targets (46 up- and 123 down-regulated) were of particular interest, since their expressions were negatively correlated (r < − 0.5) with the expression of their corresponding miRNA modulators [33]. A hierarchical clustering of the mRNA targets batched by corresponding miRNA regulators is shown in Additional file 8: Figure S7.

Although the results were likely compromised by seeding a small sample size (169 mRNA targets), we were able to map a small set of pathways. Interestingly, the core of the mined networks still shared the same family trees enriched by the global gene set of GoI-μG. The list included NGF signaling, FGF signaling, p38 MAPK signaling, HGF signaling, GM-CSF signaling, chemokine signaling, IL-4 signaling and NF-*k*B signaling.

miR-4651 (fold change: 2.21) regulated the largest number of mRNAs [37], which was 35% of the 169 significant mRNA targets.

### Pathway focused proteomic assays

Pathway-focused proteomic assays were performed to illustrate the impact of μG on LPS-induced host response (Figure 5). A more than two fold change from the background level was considered significant. Some of the proteins relevant to the μG studies, such as IL-2, IL-10 and IFN- were assayed, but failed to meet the quality control threshold, and therefore, are not reported here.

The protein profile associated with cytokine signaling included IL-8, IL-6, IL6-ra, and B2M that were all elevated, and IL-17 and IL-1ra that were suppressed in S-C. Likewise, IL-8, IL-6, IL1-ra, and B2M were elevated in G-4 h in comparison to G-C. In contrast, there was a modest elevation in IL-8 and IL-6, and suppression in IL-6ra and B2M in S-4 h as compared to S-C.

In addition, we investigated the protein profile associated with growth factors, which showed elevated HGF, PDGF, MCP-1, TIMP-1 and VEGFR-1/-2; and unchanged G-CSF and VEGF in S-C comparing G-C. In comparison to same base like, *i.e.* G-C, G-4 h showed elevated HGF, PDGF, TIMP-1, MCP-1, VEGFR-1; a modestly elevated VEGFR-2; and unchanged VEGF and G-CSF. In contrast, S-4 h in comparison to S-C depicted modestly elevated MCP-1, VEGF, PDGF, G-CSF; unchanged VEGFR-2; and suppressed HGF, TIMP-1 and VEGFR-1.

Individual proteomic assays using a 96-well plate based sandwich ELISA validated the expressions of IL-6, IL-8, VEGF, TIMP1 and MCP1/CCl2.

## Discussion

The focus of the present study is to find the molecular signatures associated with the host response to LPS mediated by μG. Our primary motivation was attributed to many evidences alleging the pathogenic interference as the major challenge to the space missions [38, 39]. Accompanying evidence supported the extraterrestrial condition as the potent habitat and stimulator of aggressive virulence [13–15, 38]. The perennial pathogenic threat may seriously intimidate the future of space missions [16]; a solution to the problem is therefore exigent.

The host in μG is likely to counter a dual challenge. The intrinsic challenge is defined by its own immune-compromised state stressed by μG, while pathogens with augmented proliferation and virulence constitute the additional challenge. As a consequence, this atypical opportunistic infection may activate novel bionetworks different from its terrestrial counterparts. The present study was designed to investigate this hypothesis and to our knowledge, this is the first of this kind of attempt, although similar studies have been performed in simulated reduced gravitational environment [22]. Comprehensive genomics and transcriptomic studies were purposed to identify key networks involved in extraterrestrial immune response. Putative markers were suggested, although robust validation was beyond the scope of the present project since we probed a small sample size. Further investigations with larger sample sizes are essential.

In the present study, human microvascular endothelial cells were exposed to LPS for 4 h and 8 h in spaceflight and on ground. The untreated bioreactors processed in both gravitational limits defined the controls for respective gravitational limits. All the assays were performed in duplicate, and concurrently in spaceflight (STS-135, Atlantis launched on July 11^th^, 2011) and on ground (Kennedy Space Center, FL). The ground study mimicked the other elements of the environment of the spaceflight, such as temperature and humidity (Figure 1). We were limited to use of only six bioreactors in the spaceflight, considerably compromising the power of the study. To overcome the study limitations, we took stringent measures to optimize every aspect of the assay designs detailed in the supplementary section.

### Transcriptomics and biological annotations

Two orthogonal gene sets were identified from the analyses of the transcriptome microarrays. We carried out a pair-wise moderated t-test routine with cut-off *p* < 0.05, a recommended variance modeling strategy for such a small sample size [31]. The larger gene set consisting of 5,379 transcripts (GoI-LPSμG) was the potential signatures of the host response to *LPS assault mediated by* μ*G*. A smaller set of 2,517 transcripts (GoI-μG) *exclusively altered by* μ*G* suggested that the reduced gravity was potentially a less significant contributor in molecular perturbations as compared to the LPS assault. This methodological decision proposed herein was guided by the hypothesis that μG, LPS assault and their interaction play major roles in perturbing the molecular makeups. The role of the supplementary factors, such as those beyond the experimental control might have been limited by designing a well-regulated experimental strategy.

The durations of LPS exposure (4 h vs. 8 h) emerged as the less significant factor on ground. G-4 h and G-8 h were positively correlated, but clustered distinctly from the ground control (G-C) depicted by a large Euclidian separation along PC 1 between them (Figure 2). Of note, the controls on the ground and in spaceflight were spatially closer since they were self-normalized to highlight the impact of gravitational forces on the host responses. A significant Euclidian separation between S-4 h and G-4 h depicted the distinctive characteristics of the host-pathogen relationship in μG. A close proximity between the S-C and S-4 h suggested a compromised *early* response to the endotoxic shock, a potential state of immune blunting. Clearly, the host cells subjected to μG induced stress failed to build resistance against the LPS assault.

Longer exposure to LPS, however, triggered a delayed response, which possibly allowed the pathogen a critical time window to evade the host resistance. As supporting evidence, immune-compromised mice (*Lbp* −/− and *CD14* −/−) succumbed to fatality caused by gram-negative bacterial infection, despite a delayed response 4 h after the bacterial invasion [40]. It is important to note that the diminished host response to LPS was only observed in the space-flown samples. Distinct clustering of G-C from G-4 h and G-8 h (Figure 2) conferred μG as a more dominant factor in mediating the immunological response, at least within the time scale probed herein. A pair of data points associated with longer LPS incubation could validate this potential explanation with higher confidence.

Using the available sample size, a comprehensive annotation pathways (Figure 3, Additional file 6: Figure S5 and Additional file 7: Figure S6, and Additional file 3: Table S3) was carried out to map the genomic members of GoI-μG and GoI-LPSμG.

Pathways co-enriched by GoI-μG and GoI-LPSμG are of the particular interest; such as the TLR pathway (Figure 3B) that plays a key role in early pathogenic recognition. In accordance to some previous reports [41] we observed an exclusive impact of μG in elevating *TLR4*, which potentially triggered the stress-induced downstream apoptosis [42]. In contrast, *Lbp* remained suppressed after both 4 h and 8 h exposure in spaceflight and on ground, which could be attributed to the high concentration of LPS administered in this study that typically inhibits TLR4 from recruiting LBP for LPS uptake [43]. The subsequent delayed elevation of *MyD88* (2.5 log_2_ fold change in G-4 h and G-8 h in comparison to G-C; −0.20 in S-4 h and 1.20 in S-8 h in comparison to S-C; Additional file 3: Table S2) and suppression of *MD-2* (2.6 fold change in G-4 h and G-8 h in comparison to G-C; 1.40 in S-4 h and −0.26 in S-8 h in comparison to S-C; Additional file 3: Table S2) further suggested a defect in recognizing LPS in spaceflight. Hereby, μG potentially inhibited the dimerization of TLR-4/MyD88 [44] and TLR4/MD-2 [45], two critical complexes tasked for early pathogenic recognition. Synergistic suppression of the LPS receptor complexes in spaceflight might have induced temporary LPS tolerance, a characteristic reminiscent of septic shock [44]. The impact cascaded towards impairing downstream apoptosis and the adaptive immune system as the transcripts encoding mitogen-activated protein kinase, TNF receptors and necrosis factors showed temporary suppression, followed by delayed activation induced by LPS insult in spaceflight (Additional file 3: Table S2).

Two other pathways of interest, namely IL-10 (Additional file 7: Figure S6A) and GM-CSF (Additional file 7: Figure S6B), were co-enriched by GoI-μG and GoI-LPSμG. The exclusive impact of μG was silencing of *IL-10* and its receptor (*IL10-Ra*), the anti-inflammatory immune regulators. In contrast, past observations reported elevated *IL-10* in space-flown murine T-cells [46]; while terrestrial UV radiation inhibited human epidermal IL-10 secretion [47].

The GM-CSF network primarily controls the production and function of blood cells and regulates a number of key apoptotic networks like PI3K/Akt pathway [48]. The transcripts encoding PI3K and AKT were elevated by μG and terrestrial LPS assault. The LPS assault in spaceflight did not enhance their activities after 4 h exposure, possibly leading to compromised cell survival and proliferation consequences.

### Novel miRNA targets

The microRNAs of the miR-200 family are classified as the redox regulators [49], and increased miR-200a and ensuing p38α depletion were reported as the consequences of oxidative stress [50]. In consideration of μG as a potential stimulant of oxidative stress primarily induced by depletion of the energy [51], miR-200a could be a putative signature of the host response to LPS assault mediated by μG. Of note, miR-200a targeted the largest pool of mRNAs from GoI-μG; in coherence, we also observed several of p38α encoding transcripts (*MAPK1*, *MAPRE2* and *MAPK14*) inhibited in S-4 h.

We observed a negative correlation between miR-146b-5p expression and the gravitational shift (expression shifted from modestly down-regulated to up-regulated as terrestrial gravity shifted to μG). Past observations suggested that there is cross talk between the suppressed miR-146a/b with the onset of cytokine signaling and TLR-mediated LPS recognition [52]. Other major microRNAs of the present study included miR-212, a signature of alcoholic stress [53] and miR-125b that was altered by *in vitro* endotoxic assault on murine cells [54].

### Pathway focused proteomics

Pathway focused proteomic investigation of a selected panel of cytokines and growth factors (Figure 5) found overexpressed IL-6 [10, 55] and IL-8 [10], and suppressed IL-17 [1] in S-C supporting various past reports. Suppressed *PDGF-*β receptor in rat osteoblast cells was observed in spaceflight [56], while we found increased expression of PDGF in epithelial cells. LPS assault surged the expression of IL-6 and IL-8; while others such as B2M, TIMP-1 MCP-1 and VEGFR failed to respond to the extraterrestrial LPS assault, potentially indicating a state of diminished immunity. Overexpression of VEGFR-1/-2 in μG could be interpreted as a protective measure against UV-induced photo-damage [57]. Terrestrial LPS shock induced the pro-survival mechanisms promoted by overexpressed VEGFRs, while the LPS shock in spaceflight failed to stimulate the same.

In the context of the alleged link of μG to bone loss [58], the synchronized activities of TIMP-1 and MMP-9 were of particular interest. Typically, TIMP-1 operates counteractively with MMPs [59]. Overexpressed TIMP-1 in spaceflight (MMP9 registered no change) could feedback control the bone-resorbing activity [37]. LPS shock augmented TIMP-1 on ground but inhibited it in spaceflight, thereby possibly restricting the regeneration of the epidermis and angiogenesis of the wound. The imbalance between TIMPs and MMPs could delay wound healing [60].

## Conclusions

The present study, although compromised by the small sample size, was able to illustrate several aspects of immunological responses to the endotoxic assault mediated by μG. It was beyond the scope of present paper to probe the pathways with non-immunological implications; although such proposition could have many scientific interests. We created the critical distinguishing features of the present study by implementing the host-pathogen interactions in spaceflight and subsequently lysing the cells onboard. Thereby the present study is set characteristically apart from the past reports. In principal, this customized technological maneuver helped us to separate the host-pathogen relationships from the terrestrial bias. The deliverables were the molecular markers *specifically* associated with host responses mediated by μG.

A synergistic immune blunting was evident in spaceflight. The early pathogenic detection in spaceflight was feeble and delayed which could confer a critical time advantage to the pathogen, facilitating an opportunistic invasion. An *in vivo* study reported fatality caused by very similar time delay in responding to endotoxic shock [40]. Downstream apoptotic response and growth factor recruitment processes were negatively affected, as well. Impaired healing and defective angiogenesis were other major proteomic findings.

MicroRNA candidates such as miR-200a and miR-146b are typically associated with oxidative stress and immune regulation, respectively, and were found altered by the LPS assault in spaceflight. A multitude of evidence suggesting μG as a compelling modulator of oxidative stress [51] and immune suppression [2, 12, 16] further intrigues our interest in these miRNA families identified herein. The significant relationships of these miRNAs to the phenotypic signatures of the extraterrestrial conditions still stands out; despite of the compromised power of the study due to the small sample size. Systematic investigation of these genomic regulators could potentially throw valuable insights into the very young and dynamic field of astrobiology. Taken together, a more comprehensive follow up assessment is necessary to validate the observations presented here. In particular, the elaborate *in vitro* assays with strategic time points beyond the time frame introduced herein and the coherent *in vivo* studies are recommended.

## Electronic supplementary material

Additional file 1: Figure S1: *The flowpaths of the CCM operated in the spaceflight and on ground.* The flowpath A was designed to support the bioreactors 1 and 2; and flowpath B was designed to support to the rest of the bioreactors [3–6] operated in spaceflight and on ground, concurrently. Each flowpath consisted of one bioreactor, one pump, one oxygen chamber (O_2_), one 70 ml media bag and one sump bag. An LPS bag was attached to the flowpath B, depicting the major difference between the two flowpaths, A and B. During the first 10 days of the space mission, the media from the media bags was circulated through the path marked by “Common” (C) and “Normally Open” (NO). On the 11^th^ day, the unidirectional “Normally Closed” (NC) paths connecting LPS bags to the bioreactors 5 and 6, respectively, were switched on. Four hours later, LPS was injected to the flowpaths integrated to bioreactors 3 and 4. After the next 4 hours, the media bags were disconnected from the flowpaths by shutting off the associated “Normally Open” (NO) paths. The LPS bags were disconnected, too, by shutting off the respective “Normally Closed” (NC) paths. At the same time, the “Normally Closed” paths linked to the RLT bags and sump bags were switched open in all flowpaths integrated to bioreactors 1 to 6. The consequent arrangement was maintained for the remaining 5 days of the space mission. We obtained nucleic acids and proteins from the bioreactors and media bags, respectively. Sump bags collected proteins, too, but not in an optimum amount to run meaningful studies. (JPEG 49 KB)

Additional file 2: Figure S2: *Optimization of LPS concentration and incubation duration*. The cell viability was measured from a series of assays probing cells exposed to a dynamic range of LPS concentrations (0.01-1000 ng/ul) for various durations (2–24 h). The x-axis corresponds to the range of LPS exposure duration and y-axis to the percentage cell viability comparing the time matched control cells. The arrows point out the parameters selected for final experiments (100 μg/ml LPS treatment for 4 h and 8 h). Hereby, two conditions showing little to modest loss of cell viability are selected with the presumption that microgravity would cause further cell damage. Optimum numbers of viable cells are required for carrying out the downstream multi-omics assays. (JPEG 45 KB)

Additional file 3: Table S1: *Identification of most suitable cell lysis and nucleic acid fixing buffer.* Six commercially available lysis buffers purchased from three vendors were tested. The integrity and concentration of RNA and DNA extracted from HUVEC-dBL cells were investigated for 15 days suspending the nucleic acids in respective buffer at room temperature. The performances of the buffer candidates were measured compared to RLT solution and ranked. The * marked entries were top 90 percentile candidates. The RNA integrities were probed in the scale of 260/280 and 260/230 ratios and the yields (μg) were used to rank their performances. **Table S2.** *Gene lists of interest.* Two gene lists are recorded herein, enriched by genes (i) altered by microgravity exclusively (GoI- μG) and (ii) altered by LPS assault mediated by microgravity (GoI-LPSμG). For respective genes the regulation of S-C, G-4 h, G-8 h (all normalized by G-C) and S-4 h and S-8 h (both normalized by S-C) were reported. **Table S3.** *Pathways of interest.* The pathways were significantly enriched by genes (i) altered by microgravity exclusively and (ii) altered by LPS assault mediated by microgravity. For respective functions the number of participating genes and percentage of enrichment were reported. The pathways were batched under three parent terms (i) cytokine signaling, (ii) apoptosis and (ii) growth factor signaling. **Table S4.** *The identified list of miRNA and corresponding mRNA targets.* The miRNA-mRNA pairs are curated herein mapping from the gene sets (i) altered by microgravity exclusively (GoI-μG) and (ii) altered by LPS assault mediated by microgravity (GoI-LPSμG). The regulations of S-C (normalized by G-C) were reported for the genomic subsets of GoI- μG. Likewise, the regulations of G-4 h (normalized by G-C) and S-4 h (normalized by S-C) were reported for the genomic subsets of GoI-LPSμG. (DOCX 245 KB)

Additional file 4: Figure S3: *The Venn diagram of the gene profiles illustrating the characteristics of the gene of interest.* The population overlaps of the three gene sets mined in the present study are depicted (not in scale by size). A pair wise moderated *t*-test identified 2,517 transcripts altered between ground and space control (G-C vs. S-C), which is depicted in the red circle in the upper left **(Set A)**. We consider this subset of 2,517 transcripts as the markers of microgravity, marked by broken yellow line and defined as GoI-μG. Similar routine identified 1,302 genes significantly altered between G-C and G-8 h assays **(Set C)**. This gene set is reported in the blue circle in the upper right. The intrinsic host response independent of the gravitational shift could be the primary factor in altering these transcriptomic expressions. Likewise, 7,879 genes altered between G-4 h and S-4 h **(Set B)**. The green circle in the lower middle depicts this transcript set. Systematically rejecting the transcripts possibly associated with intrinsic host response and microgravity exclusively, we curated 5,379 molecular signatures of host response to LPS assault mediated by μG (μG x host response to LPS insult) and defined as GoI-LPSμG. (JPEG 56 KB)

Additional file 5: Figure S4: *Hierarchical clustering of the genes significantly enriching the pathways of interest.* The Euclidian algorithm clustered the transcripts listed in three focused groups of pathways linked to cytokine signaling, apoptosis and growth factor signaling. **(A)** The right-most column represents the transcripts altered by the gravitational change (GoI-μG), segregated from top to bottom in the order of cytokine signaling, apoptosis and growth factor signaling. **(B-D)** The Euclidean clustering represents the regulation of the transcripts **(B)** encoding cytokine signaling **(C)** apoptosis and **(D)** growth factor signaling altered by LPS insult mediated by gravitational alteration (GoI-LPSμG). The assays were carried out by exposing endothelial cells to LPS for 4 and 8 h on ground (two left-most columns of **B-D**) and in the spaceflight (two right-most columns of **B-D**). (JPEG 83 KB)

Additional file 6: Figure S5: *Enrichment profile of the immunologically relevant pathways significantly altered by microgravity.* Ten most significantly enriched (*p* < 0.1) pathways are identified focusing on three parent nodes, namely cytokine signaling (3 networks) apoptosis (3 networks) and growth factor signaling (4 networks). The fractional sharing of elevated (fold change > 2) and suppressed (fold change < −2) genomic members are colored by red and green, respectively. The unchanged fractions are shaded yellow. The color scheme is in the right. (JPEG 62 KB)

Additional file 7: Figure S6: **A**. *IL-10 signaling pathway.* A schematic network of IL-10 pathway is presented. The IL-10 pathway is co-enriched by both gene sets linked to LPS insult mediated by microgravity (GoI-LPSμG) and exclusive effects of microgravity (GoI-μG), respectively. There are three blocks beneath each gene name representing the transcriptomic regulation of S-C (left most block), G-4 h (middle block) and S-4 h (right most block). Both G-8 h and S-8 h are not shown in the figure. The S-C regulations normalized by G-C (S-C/G-C) represent the exclusive impacts of microgravity. And, G-4 h and S-4 h normalized by respective controls (G-4 h/G-C and S-4 h/S-C) represents the influence of the gravitational shift on host response to LPS assault. The scale representing the color scheme is included. **B**. *GM-CSF signaling pathway.* A schematic network of the GM-CSF pathway is presented. The GM-CSF pathway is co-enriched by both gene sets linked to LPS insult mediated by microgravity (GoI-LPSμG) and exclusive effects of microgravity (GoI-μG), respectively. There are three blocks beneath each gene name representing the transcriptomic regulations of S-C (left most block), G-4 h (middle block) and S-4 h (right most block). Both G-8 h and S-8 h are not shown in the figure. The S-C regulations normalized by G-C (S-C/G-C) represent the exclusive impacts of microgravity. And, G-4 h and S-4 h normalized by respective controls (G-4 h/G-C and S-4 h/S-C) represent the influence of gravitational shift on host response to LPS assault. The scale representing the color scheme is included. (ZIP 79 KB)

Additional file 8: Figure S7: *miRNA and corresponding mRNA targets regulated by microgravity.* Nine miRNAs significantly altered by gravitational limits are clustered in the left column. There are 169 mRNAs clustered in the right column collectively targeted by these 9 miRNAs of interest. The mRNAs are batched according to their miRNA modulators. The scale representing the color scheme is included. (JPEG 33 KB)
