# Supplementary figures and images for: An integrated omics analysis: impact of microgravity on host response to lipopolysaccharide in vitro
Source: BMC Genomics. 2014 Aug 7;15(1):659. doi: 10.1186/1471-2164-15-659 (PMC4287545; doi:10.1186/1471-2164-15-659)

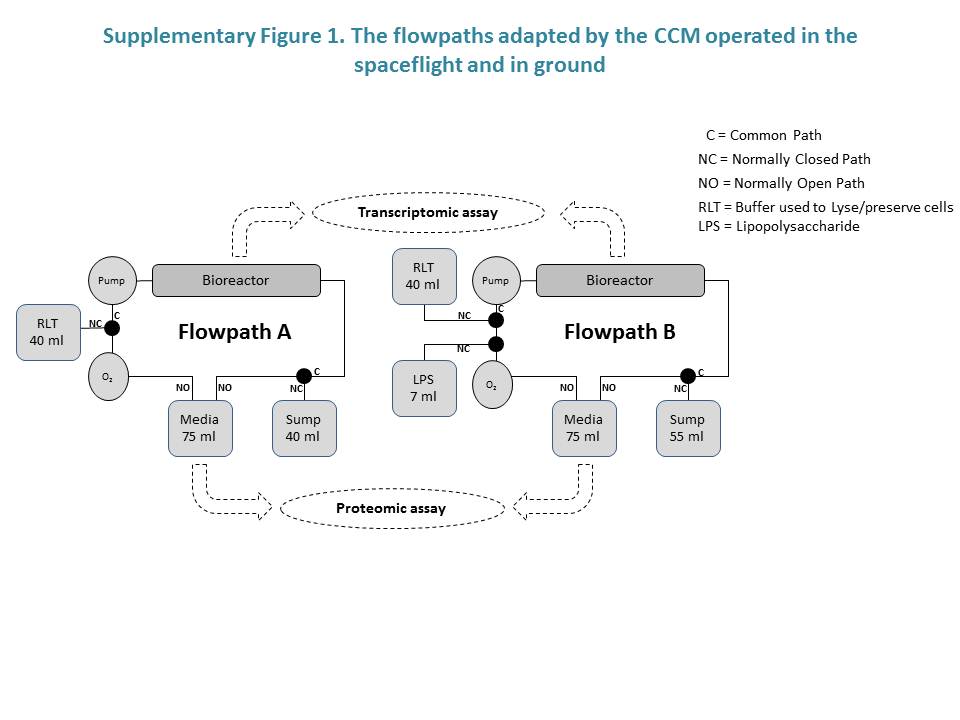

Supplement: Supplementary file 1 — Additional file 1: Figure S1: The flowpaths of the CCM operated in the spaceflight and on ground. The flowpath A was designed to support the bioreactors 1 and 2; and flowpath B was designed to support to the rest of the bioreactors [3–6] operated in spaceflight and on ground, concurrently. Each flowpath consisted of one bioreactor, one pump, one oxygen chamber (O2), one 70 ml media bag and one sump bag. An LPS bag was attached to the flowpath B, depicting the major difference between the two flowpaths, A and B. During the first 10 days of the space mission, the media from the media bags was circulated through the path marked by “Common” (C) and “Normally Open” (NO). On the 11th day, the unidirectional “Normally Closed” (NC) paths connecting LPS bags to the bioreactors 5 and 6, respectively, were switched on. Four hours later, LPS was injected to the flowpaths integrated to bioreactors 3 and 4. After the next 4 hours, the media bags were disconnected from the flowpaths by shutting off the associated “Normally Open” (NO) paths. The LPS bags were disconnected, too, by shutting off the respective “Normally Closed” (NC) paths. At the same time, the “Normally Closed” paths linked to the RLT bags and sump bags were switched open in all flowpaths integrated to bioreactors 1 to 6. The consequent arrangement was maintained for the remaining 5 days of the space mission. We obtained nucleic acids and proteins from the bioreactors and media bags, respectively. Sump bags collected proteins, too, but not in an optimum amount to run meaningful studies. (JPEG 49 KB) [file 12864_2014_6776_MOESM1_ESM.jpeg]

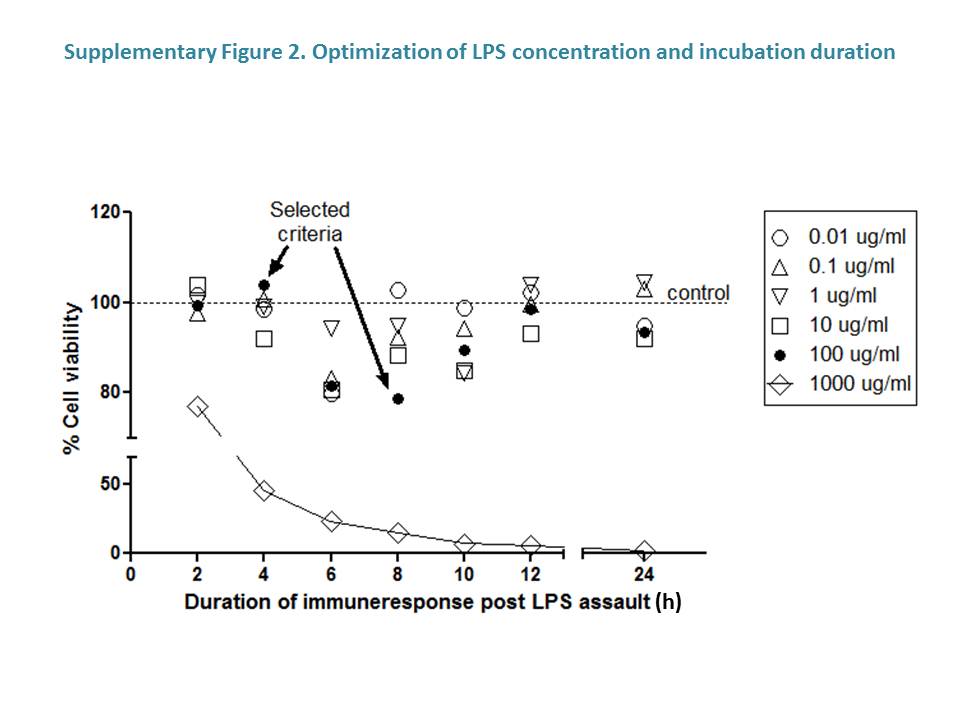

Supplement: Supplementary file 2 — Additional file 2: Figure S2: Optimization of LPS concentration and incubation duration. The cell viability was measured from a series of assays probing cells exposed to a dynamic range of LPS concentrations (0.01-1000 ng/ul) for various durations (2–24 h). The x-axis corresponds to the range of LPS exposure duration and y-axis to the percentage cell viability comparing the time matched control cells. The arrows point out the parameters selected for final experiments (100 μg/ml LPS treatment for 4 h and 8 h). Hereby, two conditions showing little to modest loss of cell viability are selected with the presumption that microgravity would cause further cell damage. Optimum numbers of viable cells are required for carrying out the downstream multi-omics assays. (JPEG 45 KB) [file 12864_2014_6776_MOESM2_ESM.jpeg]

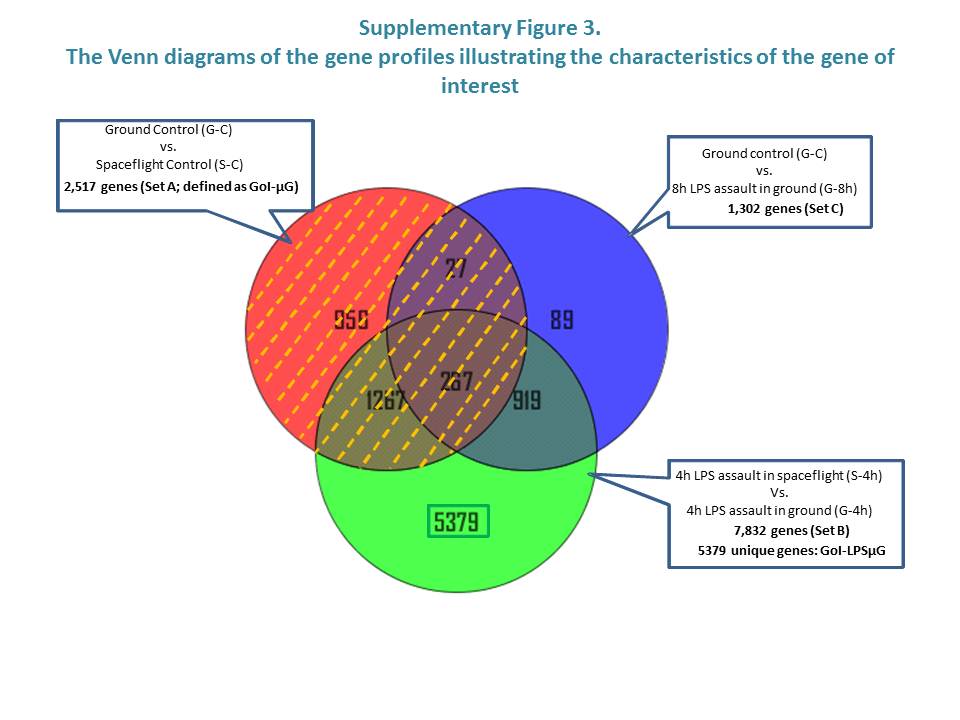

Supplement: Supplementary file 4 — Additional file 4: Figure S3: The Venn diagram of the gene profiles illustrating the characteristics of the gene of interest. The population overlaps of the three gene sets mined in the present study are depicted (not in scale by size). A pair wise moderated t-test identified 2,517 transcripts altered between ground and space control (G-C vs. S-C), which is depicted in the red circle in the upper left (Set A). We consider this subset of 2,517 transcripts as the markers of microgravity, marked by broken yellow line and defined as GoI-μG. Similar routine identified 1,302 genes significantly altered between G-C and G-8 h assays (Set C). This gene set is reported in the blue circle in the upper right. The intrinsic host response independent of the gravitational shift could be the primary factor in altering these transcriptomic expressions. Likewise, 7,879 genes altered between G-4 h and S-4 h (Set B). The green circle in the lower middle depicts this transcript set. Systematically rejecting the transcripts possibly associated with intrinsic host response and microgravity exclusively, we curated 5,379 molecular signatures of host response to LPS assault mediated by μG (μG x host response to LPS insult) and defined as GoI-LPSμG. (JPEG 56 KB) [file 12864_2014_6776_MOESM4_ESM.jpeg]

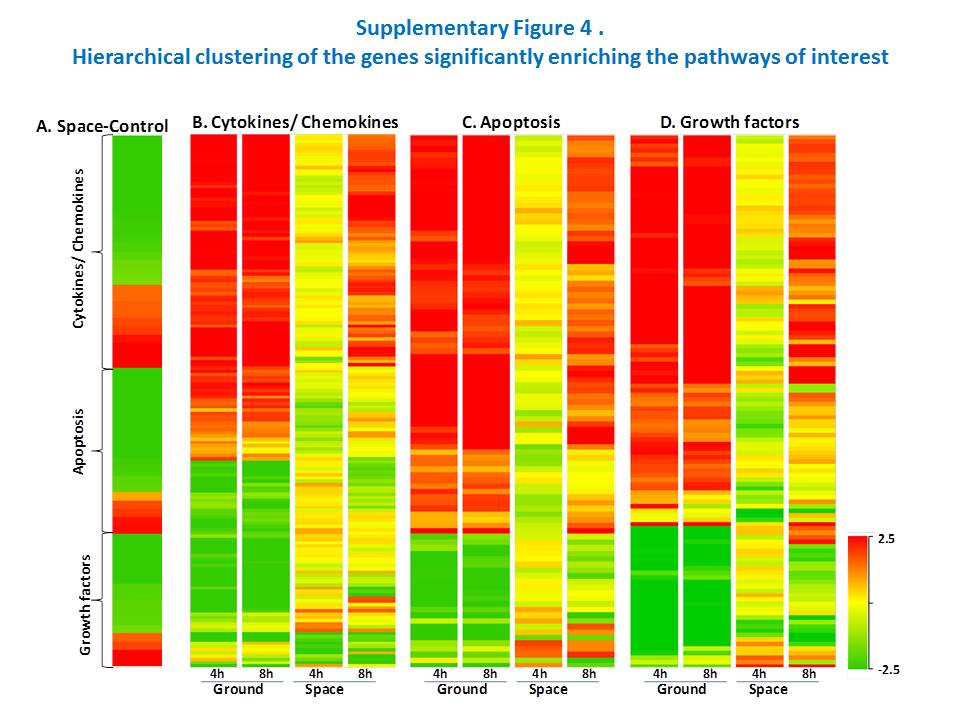

Supplement: Supplementary file 5 — Additional file 5: Figure S4: Hierarchical clustering of the genes significantly enriching the pathways of interest. The Euclidian algorithm clustered the transcripts listed in three focused groups of pathways linked to cytokine signaling, apoptosis and growth factor signaling. (A) The right-most column represents the transcripts altered by the gravitational change (GoI-μG), segregated from top to bottom in the order of cytokine signaling, apoptosis and growth factor signaling. (B-D) The Euclidean clustering represents the regulation of the transcripts (B) encoding cytokine signaling (C) apoptosis and (D) growth factor signaling altered by LPS insult mediated by gravitational alteration (GoI-LPSμG). The assays were carried out by exposing endothelial cells to LPS for 4 and 8 h on ground (two left-most columns of B-D) and in the spaceflight (two right-most columns of B-D). (JPEG 83 KB) [file 12864_2014_6776_MOESM5_ESM.jpeg]

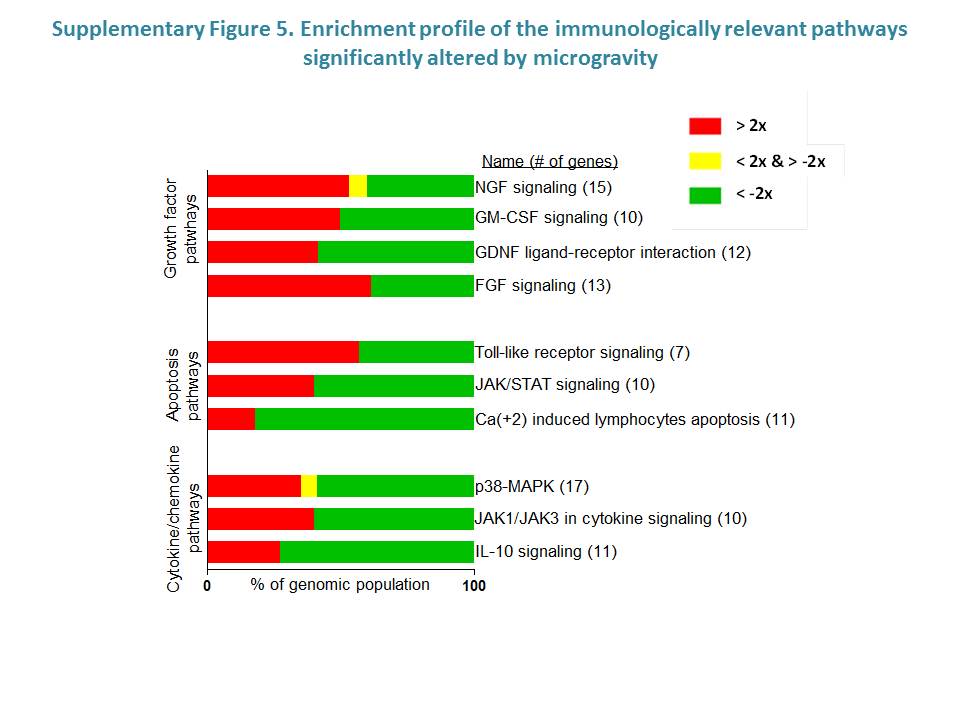

Supplement: Supplementary file 6 — Additional file 6: Figure S5: Enrichment profile of the immunologically relevant pathways significantly altered by microgravity. Ten most significantly enriched (p < 0.1) pathways are identified focusing on three parent nodes, namely cytokine signaling (3 networks) apoptosis (3 networks) and growth factor signaling (4 networks). The fractional sharing of elevated (fold change > 2) and suppressed (fold change < −2) genomic members are colored by red and green, respectively. The unchanged fractions are shaded yellow. The color scheme is in the right. (JPEG 62 KB) [file 12864_2014_6776_MOESM6_ESM.jpeg]

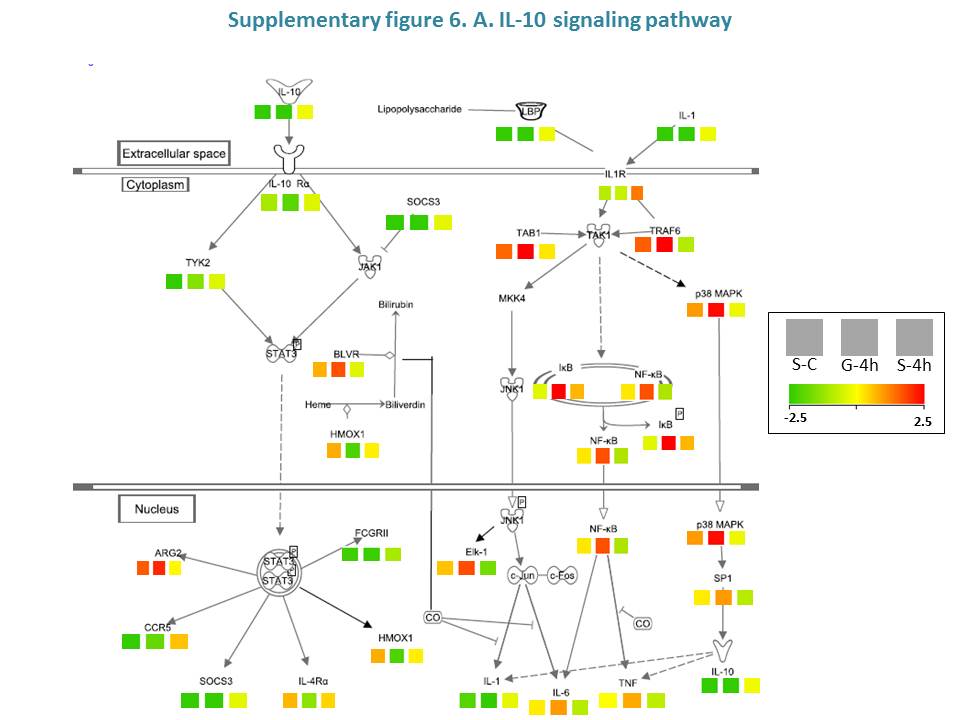

Supplement: Supplementary file 7 — Additional file 7: Figure S6: A. IL-10 signaling pathway. A schematic network of IL-10 pathway is presented. The IL-10 pathway is co-enriched by both gene sets linked to LPS insult mediated by microgravity (GoI-LPSμG) and exclusive effects of microgravity (GoI-μG), respectively. There are three blocks beneath each gene name representing the transcriptomic regulation of S-C (left most block), G-4 h (middle block) and S-4 h (right most block). Both G-8 h and S-8 h are not shown in the figure. The S-C regulations normalized by G-C (S-C/G-C) represent the exclusive impacts of microgravity. And, G-4 h and S-4 h normalized by respective controls (G-4 h/G-C and S-4 h/S-C) represents the influence of the gravitational shift on host response to LPS assault. The scale representing the color scheme is included. B. GM-CSF signaling pathway. A schematic network of the GM-CSF pathway is presented. The GM-CSF pathway is co-enriched by both gene sets linked to LPS insult mediated by microgravity (GoI-LPSμG) and exclusive effects of microgravity (GoI-μG), respectively. There are three blocks beneath each gene name representing the transcriptomic regulations of S-C (left most block), G-4 h (middle block) and S-4 h (right most block). Both G-8 h and S-8 h are not shown in the figure. The S-C regulations normalized by G-C (S-C/G-C) represent the exclusive impacts of microgravity. And, G-4 h and S-4 h normalized by respective controls (G-4 h/G-C and S-4 h/S-C) represent the influence of gravitational shift on host response to LPS assault. The scale representing the color scheme is included. (ZIP 79 KB) [file 12864_2014_6776_MOESM7_ESM.zip › add7/1949789008123310_add7a.jpeg]

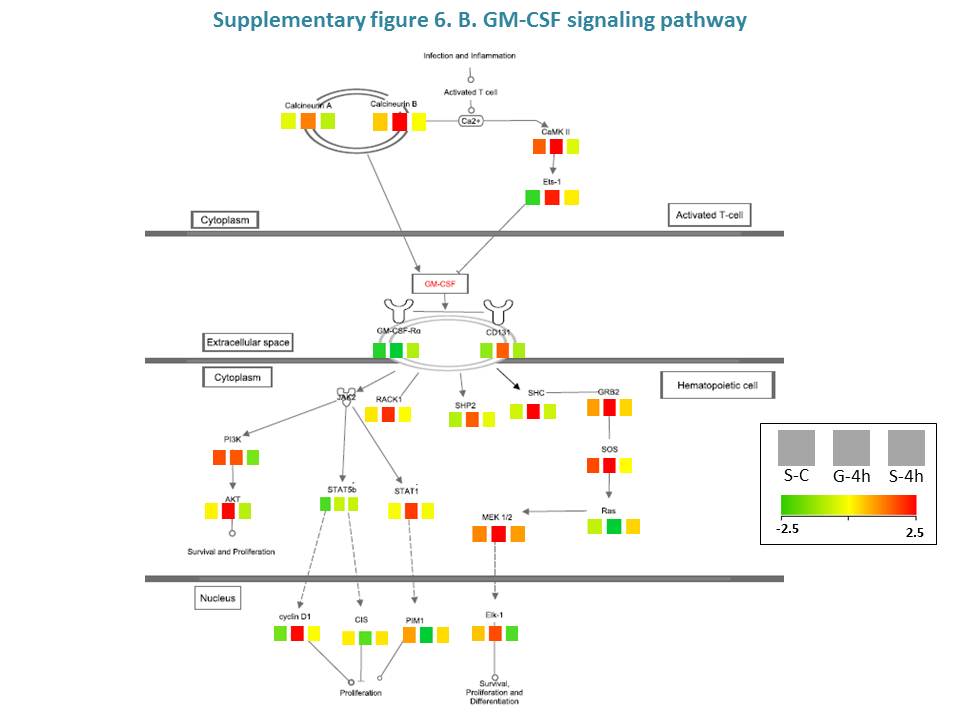

Supplement: Supplementary file 7 — Additional file 7: Figure S6: A. IL-10 signaling pathway. A schematic network of IL-10 pathway is presented. The IL-10 pathway is co-enriched by both gene sets linked to LPS insult mediated by microgravity (GoI-LPSμG) and exclusive effects of microgravity (GoI-μG), respectively. There are three blocks beneath each gene name representing the transcriptomic regulation of S-C (left most block), G-4 h (middle block) and S-4 h (right most block). Both G-8 h and S-8 h are not shown in the figure. The S-C regulations normalized by G-C (S-C/G-C) represent the exclusive impacts of microgravity. And, G-4 h and S-4 h normalized by respective controls (G-4 h/G-C and S-4 h/S-C) represents the influence of the gravitational shift on host response to LPS assault. The scale representing the color scheme is included. B. GM-CSF signaling pathway. A schematic network of the GM-CSF pathway is presented. The GM-CSF pathway is co-enriched by both gene sets linked to LPS insult mediated by microgravity (GoI-LPSμG) and exclusive effects of microgravity (GoI-μG), respectively. There are three blocks beneath each gene name representing the transcriptomic regulations of S-C (left most block), G-4 h (middle block) and S-4 h (right most block). Both G-8 h and S-8 h are not shown in the figure. The S-C regulations normalized by G-C (S-C/G-C) represent the exclusive impacts of microgravity. And, G-4 h and S-4 h normalized by respective controls (G-4 h/G-C and S-4 h/S-C) represent the influence of gravitational shift on host response to LPS assault. The scale representing the color scheme is included. (ZIP 79 KB) [file 12864_2014_6776_MOESM7_ESM.zip › add7/1949789008123310_add7b.jpeg]

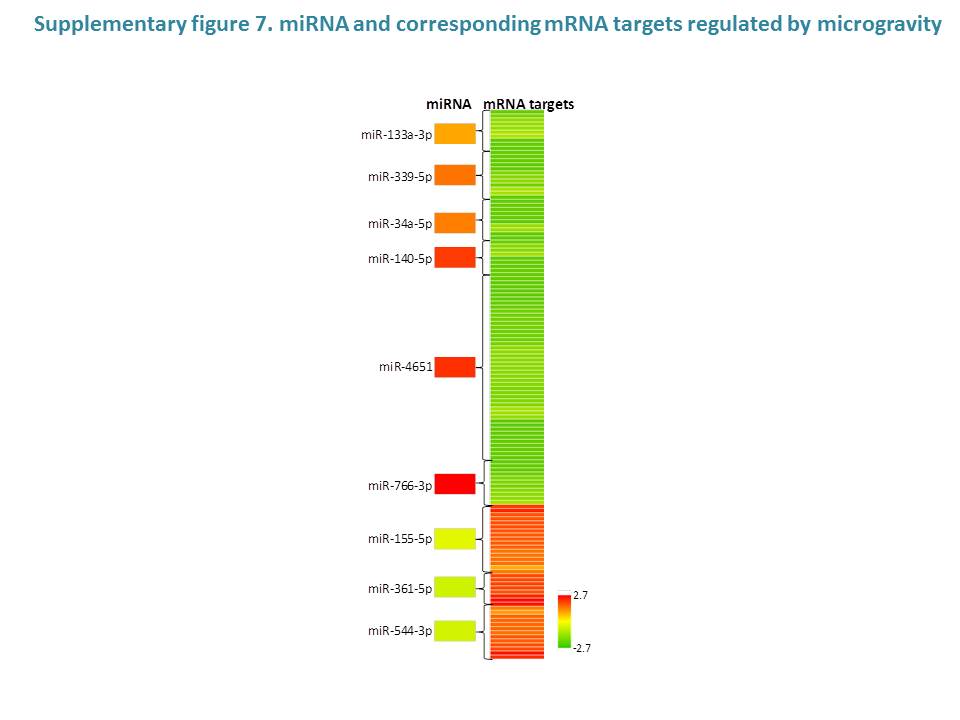

Supplement: Supplementary file 8 — Additional file 8: Figure S7: miRNA and corresponding mRNA targets regulated by microgravity. Nine miRNAs significantly altered by gravitational limits are clustered in the left column. There are 169 mRNAs clustered in the right column collectively targeted by these 9 miRNAs of interest. The mRNAs are batched according to their miRNA modulators. The scale representing the color scheme is included. (JPEG 33 KB) [file 12864_2014_6776_MOESM8_ESM.jpeg]
